# Comparison of the prevalence of probably postpartum depression before and during the covid-19 pandemic in Turkey: a systematic review and meta-analysis

**DOI:** 10.1007/s00127-025-02905-4

**Published:** 2025-04-23

**Authors:** Zekiye Karaçam, Pirozhan Ekin, Hilal Bal Şaraldı

**Affiliations:** 1 Faculty of Health Sciences, Division of Midwifery, Aydın Adnan Menderes University, Aydın, Turkey; 2 Institute of Health Sciences, Division of Midwifery, Aydın Adnan Menderes University, Aydın, Turkey

**Keywords:** COVID-19, Meta-analysis, Mothers, Prevalence, Probably postpartum depression, Women’s health

## Abstract

**Purpose:**

To determine the prevalence of probably postpartum depression and the effect of COVID-19 pandemic on the prevalence of probably postpartum depression based on the results of the studies in Turkey.

**Methods:**

Systematic review and meta-analysis of cross-sectional studies. The key words postpartum depression or postnatal depression and Turkey were searched in the electronic databases of PubMed, EbscoHost, OVID Journals, Science Direct, Web of Science, ULAKBIM Databases, DergiPARK, TR Dizin, YÖK-Natural Thesis Centre. The systematic review was performed by following PRISMA and COSMOS-E. Data were collected by using a data extraction tool developed by the researchers. The quality of the studies was evaluated by utilizing The Joanna Briggs Institute’s Critical Appraisal Checklist for Analytical Cross Sectional Studies. Obtained data were synthesized with meta-analysis, narrative synthesis, subgroup analysis and meta-regression.

**Results:**

The total sample size of 34 studies included in this meta-analysis was 10 236. The cut-off score for the EPDS was considered as ≥ 13 in 30 studies and ≥ 12 in four studies. The pooled probably postpartum depression prevalence was 17.8% (95% CI: 0.153–0.206; 95% Prediction Interval: 0.070–0.383). It was found to be 16.3% before the pandemic (95% CI: 0.065–0.358; 95% Prediction Interval: 0.065–0.358) and increased to 20.2% during the pandemic (95% CI: 0.068–0.468; 95% Prediction Interval: 0.068–0.468), though the difference was not significant (Q = 1.77; df: 1; *p* = 0.184). The meta-regression analysis showed that the prevalence of probably postpartum depression did not change depending on the geographical region where the studies were performed, the time of data collection and the cut-off point of the EPDS. However, the studies reported many factors related to women, their infants and families that affected the prevalence of probably postpartum depression.

**Outcomes:**

This meta-analysis revealed that the prevalence of probably postpartum depression was very high, increased during the pandemic and was affected by many risk factors. It may be recommended that healthcare professionals take protective and improving measures for the mental health of women at high risk during the perinatal period and provide early diagnosis, treatment, monitoring and care services.

**Supplementary Information:**

The online version contains supplementary material available at 10.1007/s00127-025-02905-4.

## Introduction

A new type of corona virus disease (COVID-19) caused by SARS-CoV-2 quickly spread throughout the world. Infecting 761 071 826 people and causing 6 789 677 deaths by June 2023, SARS-CoV-26 caused a global health crisis [1]. One of its global effects is that women experienced more mental health problems [2, 3]. Rapid spread of COVID-19 affected how women received care during pregnancy and birth and after birth. One health problem more frequently experienced by women during the pandemic is postpartum depression (PPD) [2, 4–6]. PPD affects maternal and infant health, family health, the quality of life, infant nutrition, growth and development [7, 8].

According to the Diagnostic and Statistical Manual of Mental Disorders, Fifth Edition, a major depressive episode with peripartum onset is defined as PPD when it occurs during pregnancy or up to four weeks after delivery [9]. PPD is usually characterized by temporary mood swings, sleeplessness, disorganized behavior, nervousness and agitation [10]. Besides, the feeling of guilt and helplessness, hostility, inability to concentrate, introversion, loneliness, fear, loss of control, fear of becoming insane, suicidal ideation, lack of sexual drive, tendency to cry, stress, self-isolation and apathy to the infant may appear [8, 11]. Restricted access of postpartum women to health professionals due to measures against COVID-19 taken in health centers caused the women to be alone and the prevalence of PPD symptoms to increase [12, 13].

During the postpartum period, mothers may experience intense feelings of happiness with negative mood symptoms. Maternity blues, which is frequently experienced during this period and is seen in approximately 80% of mothers, should be distinguished from PPD. Maternity blues is a condition that shows various symptoms such as emotional lability, irritability and tearfulness that begin in the first few days after birth and continue until the second week. Maternity blues can be managed with supportive care such as adequate sleep, rest, and emotional and physical support. If symptoms are severe, enough to affect daily functioning or last longer than two weeks, the mother should be evaluated for postpartum depression and anxiety [14, 15].

There have been several comprehensive meta-analyses on the prevalence of probably PPD. Globally, the prevalence of probably PPD ranges from 11.5 to 27% in the meta-analyses including studies performed before the pandemic [7, 10, 16–24]. In meta-analysis studies based on information produced in Turkey, the prevalence of probably postpartum depression in the pre-pandemic period was reported to be 24% [18, 19]. It varies between 26.7% and 34% in the meta-analyses including studies performed during the pandemic [2, 4–6]. Clearly, the prevalence of probably PPD is relatively higher among women during COVID-19 pandemic.

Many psychosocial, cultural and biological risk factors before, during and after birth have been associated with the etiology of PPD. Among these factors are age of the mother at the time of delivery [25], being married, a lack of autonomy [25], primiparas [22], multiparity [26], unplanned pregnancy [23, 25, 27], stress and anxiety [4, 28], inadequate social support [4, 23, 25, 27], violence at home [23, 25], lack of satisfaction with marriage [23], a poor relationship [27], history of abortion [28], death of infant [23], low financial status [7, 27], pregnancy-related complications [16], diabetes mellitus in pregnancy [20], epidural anesthesia during delivery [20], loss of autonomy about delivery [29], a poor obstetric condition [7], breastfeeding problems [26, 29], prior history of depression [7, 23], depression during pregnancy [20], having a boy baby [20] and history of adverse birth and infant health outcomes [7].

Maternal-infant and family healthcare needs increase in the postpartum period. Isolation due to COVID-19 pandemic caused failure to meet these needs and triggered PPD [30, 31]. The WHO recommends that PPD should be decreased and that mental healthcare services should be incorporated into available maternal healthcare services to achieve it [32]. Therefore, health professionals performing follow-up and care of mothers and their infants should continue to offer preventive care, early diagnosis, follow-up and care services regarding PPD [8, 33]. Midwives and nurses should adopt a whole person approach when dealing with women experiencing PPD symptoms and provide guidance for their families about how to cope with it [12, 17, 34].

There have been meta-analysis on the prevalence of probably PPD in several countries including Turkey before and during COVID-19 pandemic. Unlike prior meta-analyses, the present meta-analysis aimed to determine the effect of COVID-19 pandemic on probably PPD based on the most recent data. Besides, this meta-analysis was directed towards obtaining the most recent, widespread prevalence of probably PPD based on the results of cross-sectional studies in Turkey and to reveal the change over time by comparing the results of prior meta-analyses. Results of the present meta-analysis will contribute to accumulation of scientific knowledge about the issue and provision of appropriate maternal-infant and family health services at both national and international levels.

### Aim and research questions

This systematic review and meta-analysis aimed to reveal the prevalence of probably PPD and the effect of COVID-19 pandemic on the prevalence of probably PPD based on the results of cross-sectional studies performed in Turkey. Answers to the following questions were sought: (1) What was the prevalence of probably PPD before and during COVID-19 pandemic? (2) Did COVID-19 pandemic affect the prevalence of probably PPD?

## Methods

### Study design

This systematic review and meta-analysis followed The Preferred Reporting Items for Systematic Reviews and Meta-Analyses (PRISMA) checklist [35] and Conducting Systematic Reviews and Meta-Analyses of Observational Studies of Etiology (COSMOS-E) checklist [36] to design the study protocol and write the article. The study protocol was registered on PROSPERO with the registry number of CRD42022376896.

### Eligibility criteria

The studies considered eligible to include in this systematic review and meta-analysis fulfilled the following PEOS criteria: Patient (P): Women experiencing the postpartum months 0–24. Exposure (E): Experiencing PPD and COVID-19 pandemic. Outcomes (O): The prevalence of probably PPD before and during the pandemic and PPD risk factors. Study design (S): Community-based cross-sectional studies conducted using the Edinburgh Postnatal Depression Scale (EPDS) in the three years before the beginning of the pandemic (from 2017 to 2019) and during the pandemic (from 2020 to 2022) in Turkey, published in English and Turkish were included. In order to ensure a more balanced distribution of the data to be used in the study to compare the prevalence of probably PPD before the pandemic and during the pandemic period, the screening time before the pandemic was determined to be the period from the beginning of the pandemic to the time the study was conducted. Additionally, to ensure that this comparison was made based on more homogeneous data, only studies using EPDS and with an EPDS cut-off point of 12/13 were included in the meta-analysis. As the grey literature, relevant dissertations were also included. Exclusion criteria were failure to report the probably PPD prevalence and supply the time of data collection, conduction of the study before 2017 and use of scales other than the EPDS to determine the prevalence of probably PPD.

### Information sources and search strategy

The studies that would be included in this systematic review and meta-analysis were screened using the key words “postpartum depression or postnatal depression and Turkey” on the electronic databases of PubMed, EbscoHost, OVID Journals, Science Direct, Web of Science, ULAKBIM Databases, DergiPARK, TR Dizin, YÖK-Natural Thesis Centre on 1–15 December 2022 and screened again on 15–20 March 2023. The search strategy is given in the supplementary material Table 1. For additional searches, the reference lists of studies included in this systematic review and previously published systematic reviews were systematically checked independently by two researchers and searched via the Google Scholar website.

### Selection process of the studies

The studies included in this systematic review and meta-analysis were independently selected by two researchers using the eligibility criteria (PE and HBŞ). After repeatedly included studies were removed, titles, abstracts and the main texts of the remaining studies were examined to determine the eligible studies. Studies determined by both researchers were compiled in a session with the principal researcher. Studies on which these researchers could not reach a common opinion were re-examined considering the inclusion criteria and a consensus was reached through discussion. In order to provide access to more suitable studies in systematic review studies, it is recommended that disagreements be resolved through discussion and, when necessary, expert opinion methods [35]. Therefore, in this study, disagreements between researchers were resolved through discussion and agreement between authors was not calculated.

### Data extraction process

A data extraction tool created by the researchers was utilized. The tool was directed towards obtaining data about the researchers conducting the studies, publication year and database, date of data collection, study design, sources of data, sample size and main findings (the prevalence of probably PPD and depression-related risk factors as described in the studies). The data independently extracted by two researchers were checked and recorded on a single data extraction tool by the same researchers and rechecked by another researcher.

### Evaluation of the methodological quality of the studies

The Critical Appraisal Checklist for Analytical Cross-Sectional Studies developed by The Joanna Briggs Institute was used to evaluate the methodological quality of the cross-sectional studies included in this study [37]. The checklist is composed of eight questions and requires marking the following options to respond to the questions: “yes”, “no”, “unclear” and “inapplicable”. In the present study, the quality of a study was considered as poor when the response was “yes” to less than 50% of the questions, moderate when the response was “yes” to 51-80% of the questions and good when the response was “yes” to more than 80% of the questions. The quality of the studies was evaluated independently by two researchers. Another researcher checked their evaluations. All three researchers discussed conflicting responses and reached an agreement to create a single version of the evaluations.

### Synthesis methods of the data

The findings about the prevalence of probably PPD reported in the studies included in this systematic review were synthesized through a meta-analysis and findings about the factors affecting the prevalence were synthesized through narrative synthesis. Estimated ratios and 95% confidence interval (CI) were calculated for the prevalence of probably PPD before and during the pandemic. Comprehensive Meta-Analysis V4 was utilized for meta-analysis [38]. Cochran Q and Higgins I² were used to analyze heterogeneity between the studies. I^2^ at 0-40% indicated unimportant heterogeneity, I^2^ at 0-60% moderate heterogeneity, I^2^ at 50–90% substantial heterogeneity and I^2^ at 75-100% considerable heterogeneity (39). In the present meta-analysis, I² was higher than 50% indicating considerable heterogeneity. Therefore, results of the random effect model were taken into consideration [40–42].

A meta-regression was performed to determine the effects of the regions where the studies were conducted, time of data collection elapsing after birth and the cut-off point of the data collection tool on the prevalence of probably PPD. Besides, subgroup analyses were made to determine the prevalence of probably PPD before and during the pandemic. All the tests used were two-tailed and *p* < 0.05 was considered as significant.

## Results

### Study selection results

**Fig. 1:**
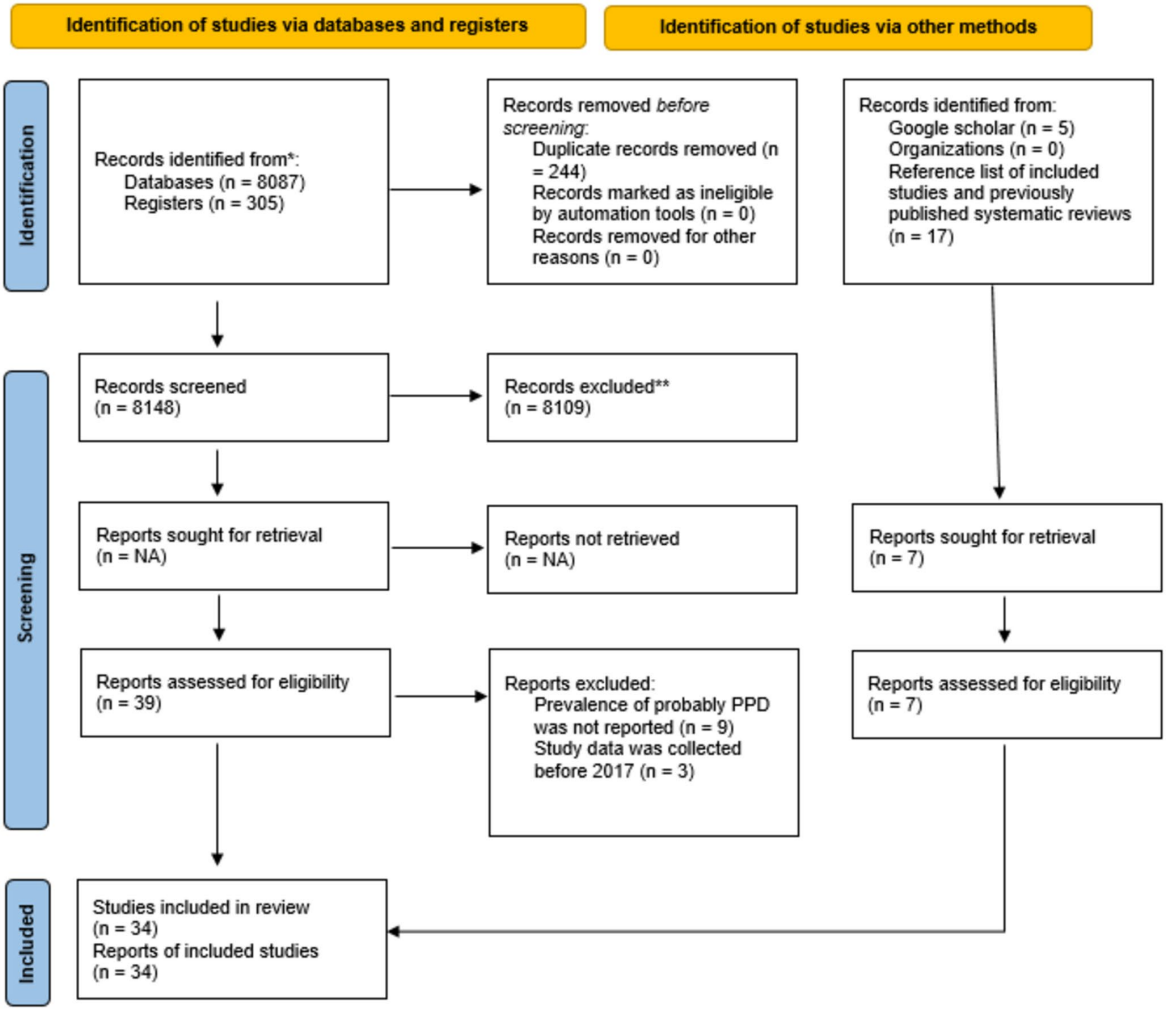
Flowchart of the study

A total of 8392 studies were found in the database searches. After the removal of duplicate studies, the titles and abstracts of the studies were examined and 39 studies were selected for full-text evaluation. As a result of the examination of these studies, 27 studies reporting the prevalence of probably PPD were selected. In addition, 7 studies reporting the prevalence of probably PPD were selected through additional searches. As a result, a total of 34 studies were included in the meta-analysis (Fig. 1). In searches conducted with the keywords of the study, the records retrieved in the databases were ranked from the most relevant to the least relevant. In this study, a large number of records obtained in the initial searches did not meet the eligibility criteria and therefore, a small number of records were selected to be assessed for eligibility.

### Characteristics of the studies and participants

All the studies included in this metal-analysis had a cross-sectional design. Data were collected before the pandemic in 22 studies, during the pandemic in 11 studies and both before and during the pandemic in one study [43].

Out of 34 studies, 10 were conducted in Marmara Region [43–52], 10 in Middle Anatolia Region [53–62], four in Black Sea Region [63–66], four in Aegean Region [67–70], two in Southeast Region [71, 72] and four in other regions [73–76]. The EPDS was used to collect data in all the studies. The cut-off point for the EPDS was considered as ≥ 13 in 30 studies and ≥ 12 in four studies [49, 64, 68, 75] (Table 1).

**Table 1:** Characteristics of included studies and main data

Authors (year) / City	Year of data collection	Study setting	Sample size	Average age of mother	Data collection tools / Cut-off score	Number of women with probably PPD / postpartum period	Related factors
Akalin et al. [[63]] / Duzce	2020	Hospital	133	30.74 ± 4.89 (20–43)	EPDS / ≥ 13	44 / 4–9 months	The fear of COVID-19.
Akbas Gunes, [[53]] / Eskişehir	2018	Family health centre	220	30.84 ± 6.032 (21–34)	EPDS / ≥ 13	35 / 2–6 months	Complications in the baby during childbirth, Smoking Physical and psychological violence during pregnancy.
Aksit, [44] / Kocaeli	2017	Hospital	113	20–44	EPDS / ≥ 13	16 / 1–4 months	No information.
Aygor and Metin, [54] / Konya	2019	Family health centre	240	28.69 ± 5.09 (19–42)	EPDS / ≥ 13	45 / 1–24 months	Feeding the baby with formula and complementary food.
Baser, [45] / Kocaeli	2018	Internet	511	17–48	EPDS / ≥ 13	102 / 1–24 months	Breastfeeding failure, living in an extended family, low education level, being a housewife, unplanned pregnancy.
Bay and Sayiner, [55] / Konya	2018–2019	Family health centre	550	--	EPDS / ≥ 13	139 / 1 month	Perception of traumatic childbirth.
Bayri Bingol and Demiroz Bal, [46] / İstanbul	2018	Family health centre	481	28.57 ± 4.92	EPDS / ≥ 13	117 / 6 months	Living in an extended family, history of abuse, low income, psychiatric treatment and miscarriage history, unplanned pregnancy, health problems during pregnancy, inadequate intrapartum care, inability to breastfeed for the first hour after birth, urinary catheterization at birth, and emergency caesarean section.
Cankaya, [56] / Konya	2019	Hospital	245	27.3 ± 5.2	EPDS / ≥ 13	44 / 2 months	Marital problems, emotional abuse, violence, sad event in the family, perceived stress and anxiety.
Cankaya and Alan Dikmen, [57] / Konya	2021	Hospital	337	28.5 ± 4.6	EPDS / ≥ 13	55 / 1–12 months	Unemployment of the spouse, low income perception, psychological and poor marital relationship, communication problems, physical and emotional violence from the spouse.
Çankaya and Ataş, [58] / Konya	2021–2022	Hospital	268	28.9 ± 5.7	EPDS / ≥ 13	71 / 1–12 months	Insufficient antenatal care, psychological problems during pregnancy and postpartum, exposure to emotional violence, having low emotional intelligence.
Comert and Bayri Bingol, [47] / İstanbul	2018	Hospital	280	24.75 ± 4.75	EPDS / ≥ 13	65 / 1 month	Inability to exclusively breastfeeding, domestic violence, health problems during pregnancy, vaginal examination by more than one person at birth.
Dagli et al. [73] / Adana	2017	Family health centre	123	18–42	EPDS / ≥ 13	40 / 3–12 months	Not having an official ending, being a housewife, low income level and living with an extended family, having unhealthy baby, assisted birth, having postpartum problems, poor marital relationship, and sexual dysfunction.
Erten et al. [67] / Kutahya	2021	Hospital	178	27.80 ± 5.24	EPDS / ≥ 13	31 / 0–12 months	Having guests at home.
Gok et al. [68] / Denizli	2020–2021	Hospital	318	--	EPDS / ≥ 12	104 / 2 months	Low education levels mothers and their partners, depression history, dissatisfaction from marriage, smoking, increased number of parity and abortion, unplanned pregnancy, nausea and emesis during pregnancy, heath problem of baby, caesarean, insufficient support and lack of breastfeeding.
Guvenc et al. [59] / Ankara	2020	Internet	212	29.49 ± 5.01	EPDS / ≥ 13	72 / 1–2 months	Presence of positive Covid-19 cases in the environment, working, high anxiety, fear of infecting the baby with Covid-19.
Ilter Bahadur et al. [71] / Diyarbakır	2019	Hospital	900	26.94 ± 5.6	EPDS / ≥ 13	90 / 1 month	Low income, history of postpartum depression, unwanted pregnancy, perceived sufficient antenatal care, history of childhood emotional and physical abuse and neglect, incarcerated household member, lack of social support.
Kahveci, [72] / Diyarbakır	2018	Hospital	311	27.5 ± 5.9 (18–43)	EPDS / ≥ 13	47 / 1–12 months	Advanced maternal and spouse age, four or more pregnancies, history of miscarriage, 3 or more living children, unplanned pregnancy, history of pre-pregnancy depression, caesarean delivery, desire for baby boy, not breastfeeding, not giving adequate care to the baby, poor marital relationship, insufficient family support, low income level, health personnel’s decision on the mode of delivery, having unhealthy baby.
Konus, [48] / Edirne	2019–2020	Family health centre	403	28.25 ± 4.99 (19–44)	EPDS / ≥ 13	101 / 1–12 months	Age 25 and younger, low education level, low education level of the spouse and being a worker, social security, low income level, living in an extended family, smoking, having 3 or more pregnancies, history of miscarriage and premenstrual syndrome, unplanned pregnancy, depression during pregnancy and history of health problems, not attending childbirth preparation class.
Korkmaz and Yilar Erkek, [64] / Giresun	2018	Family health centre	207	29.02 ± 4.87 (19–41)	EPDS / ≥ 12	38 / 1–12 months	Attachment, patience and tolerance to the baby.
Ors, [49] / Kocaeli	2017	Hospital	324	18–48	EPDS / ≥ 12	39 / 1–3 months	Being at an advanced age (over 41), a history of mental illness, not breastfeeding, inability to breastfeed only, smoking.
Oskovi Kaplan et al. [60] / Ankara	2020	Hospital	223	Median: 26 (9) years	EPDS / ≥ 13	33 / 1 month	Low maternal attachment.
Ozdemir, [50]] / İstanbul	2019–2020	Family health centre	193	18–45	EPDS / ≥ 13	14 / 2–12 months	Being over 40 years old, working, low education and income level, history of chronic disease, abortion, stillbirth, having high risk pregnancy, history of psychiatric problems, unplanned and unwanted pregnancy, low birth weight of the baby, health problem in the mother at birth, poor marital relationship.
Palancı and Aktaş, [65] / Trabzon	2018–2019	Family health centre	254	29.44 ± 5.29	EPDS / ≥ 13	54 / 2–6 months	Self-care, maternal psychology, infant care, social support, adaptation to the maternal role.
Pamuk and Guclu, [69] / Izmir	2020–2021	Family health centre	302	29.49 ± 5.18 (19–44)	EPDS / ≥ 13	44 / 1–18 months	History of personal and familial psychiatric disorders, chronic disease, poor communication with the partner, partner’s irregular employment status, psychiatric disorders during the previous birth, inadequate prenatal care, and lack of a helper in baby care.
Sahin, [70] / İzmir	2017	Hospital	255	17–45	EPDS / ≥ 13	78 / 2–12 months	Low education level, unwanted pregnancy, and smoking.
Sahin and Seven, [74] / 23 cities in Turkey	2017–2018	Hospital	497	27.79 ± 5.67	EPDS / ≥ 13	24 / 0–6 months	Mean age of women and their husbands, duration of marriage, parity, having friendship and family support, and history of receiving professional psychological support.
Sezer, [51] / Sakarya	2020 − 2021	Family health centre	282	29.70 ± 5.38 (18–43)	EPDS / ≥ 13	39 / 1–2 months	Low education, obtaining information from social media about working, spouse’s unemployment, working, family history of psychiatric illness, smoking, and health problems during pregnancy, emergency caesarean section, inadequacy and lack of support on baby care, lack of husband support, domestic violence, not exercising.
Sunay et al. [75] / Malatya	2018	Hospital	381	28.20 ± 5.63 (17–47)	EPDS / ≥ 12	54 / 0–3 months	Personality traits (responsibility, agreeableness, emotional stability).
Tug, [61] / Ankara	2018	Family health centre	222	--	EPDS / ≥ 13	49 / 2 months	Emergency caesarean section, having a daughter, not breastfeeding in the first hour after birth, unplanned pregnancy, 4 or more pregnancies, advanced maternal age (35 years and above).
Turkeli, [66] / Ordu	2020	Hospital	300	19 years and older	EPDS / ≥ 13	32 / 0–12 months	Low income, unplanned pregnancy, use of induction in labour, prolonged labour, lack of support and self-care.
Turkgeldi and Yildiz, [43] / İstanbul	2020	Hospital	Before pandemic: 73 During pandemic: 91	--	EPDS / ≥ 13	Before pandemic: 8 During pandemic: 8 / 1 month	No information.
Ugurlu et al. [62] / Ankara	2019	Hospital	300	29.02 ± 5.92	EPDS / ≥ 13	24 / 1 month	Level of breastfeeding self-efficacy, satisfaction with the relationship with their spouse, employment status of the spouse.
Yaksi and save, [52] / İstanbul	2020	Family health centre	303	--	EPDS / ≥ 13	30 / 0–6 months	Having health problem during pregnancy, unplanned and unwanted pregnancy, smoking and stressful event during pregnancy, lack of social support, history of psychiatric illness, loss, poor marital relationship, low economic level, primiparity, low birth weight.
Yilmaz et al. [76] / İstanbul	2021	Internet	206	30.67 ± 4.89 (19–45)	EPDS / ≥ 13	67 / 0–12 months	Back to work.

*

The total sample size of the studies included in this systematic review is 10 236 (min: 113-max: 900). In the studies carried out before the pandemic, data collection was performed between 2017 and 2019 and they were published between 2019 and 2020. In the studies carried out during the pandemic, data were collected between 2020 and 2023 and they were published between 2021 and 2023 (Table 1).

The participants in the studies included in this systematic review were aged 17–47 years. All of these women were white women who resided permanently in Turkey. It was determined that most of the women had primary and high school education, lived in urban areas, were housewives, defined their income level as medium, their pregnancies were planned, they gave birth 1–2 times, and caesarean births were quite common (Supplementary material Table 2).

### Results of bias risk assessment of the studies

The bias risk assessment showed that all the studies had a good quality. However, 24 studies did not have strategies to manage confounding factors (Table 2).

**Table 2:** Critical appraisal of studies

Studies	Questions of JBI Critical Appraisal Checklist For Analytical Cross-sectional Studies								Study Score
	Q1	Q2	Q3	Q4	Q5	Q6	Q7	Q8	
Akalin et al. [63]	Y	Y	Y	Y	Y	Y	Y	Y	Good (%100)
Akbas Gunes, [53]	Y	Y	Y	Y	Y	N	Y	Y	Good (%87.5)
Aksit, [44]	Y	Y	Y	Y	Y	N	Y	Y	Good (%87.5)
Aygor and Metin, [54]	Y	Y	Y	Y	Y	N	Y	Y	Good (%87.5)
Baser, [45]	Y	Y	Y	Y	Y	N	Y	Y	Good (%87.5)
Bay and Sayiner, [55]	Y	Y	Y	Y	Y	N	Y	Y	Good (%87.5)
Bayri Bingol and Demiroz Bal, [46]	Y	Y	Y	Y	Y	N	Y	Y	Good (%87.5)
Cankaya, [56]	Y	Y	Y	Y	Y	Y	Y	Y	Good (%100)
Cankaya and Alan Dikmen, [57]	Y	Y	Y	Y	Y	Y	Y	Y	Good (%100)
Cankaya and Atas, [58]	Y	Y	Y	Y	Y	Y	Y	Y	Good (%100)
Comert and Bayri Bingol, [47]	Y	Y	Y	Y	Y	N	Y	Y	Good (%87.5)
Dagli et al. [73]	Y	Y	Y	Y	Y	N	Y	Y	Good (%87.5)
Erten et al. [67]	Y	Y	Y	Y	Y	N	Y	Y	Good (%87.5)
Gok et al. [68]	Y	Y	Y	Y	Y	N	Y	Y	Good (%87.5)
Guvenc et al. [59]	Y	Y	Y	Y	Y	N	Y	Y	Good (%87.5)
Ilter Bahadur et al. [71]	Y	Y	Y	Y	Y	Y	Y	Y	Good (%100)
Kahveci, [72]	Y	Y	Y	Y	Y	N	Y	Y	Good (%87.5)
Konus, [48]	Y	Y	Y	Y	Y	N	Y	Y	Good (%87.5)
Korkmaz et al. [64]	Y	Y	Y	Y	Y	N	Y	Y	Good (%87.5)
Ors, [49]	Y	Y	Y	Y	Y	N	Y	Y	Good (%87.5)
Oskovi-Kaplan et al. [60]	Y	Y	Y	Y	Y	N	Y	Y	Good (%87.5)
Ozdemir, [50]	Y	Y	Y	Y	Y	N	Y	Y	Good (%87.5)
Palancı and Aktaş, [65]	Y	Y	Y	Y	Y	N	Y	Y	Good (%87.5)
Pamuk and Guclu, [69]	Y	Y	Y	Y	Y	Y	Y	Y	Good (%100)
Sahin, [70]	Y	Y	Y	Y	Y	N	Y	Y	Good (%87.5)
Sahin and Seven, [74]	Y	Y	Y	Y	Y	N	Y	Y	Good (%87.5)
Sezer, [51]	Y	Y	Y	Y	Y	N	Y	Y	Good (%87.5)
Sunay et al. [75]	Y	Y	Y	Y	Y	Y	Y	Y	Good (%100)
Tug, [61]	Y	Y	Y	Y	Y	N	Y	Y	Good (%87.5)
Turkeli, [66]	Y	Y	Y	Y	Y	Y	Y	Y	Good (%100)
Turkgeldi and Yildiz, [43]	Y	Y	Y	Y	Y	N	Y	Y	Good (%87.5)
Ugurlu et al. [62]	Y	Y	Y	Y	Y	Y	Y	Y	Good (%100)
Yaksi and Save, [52]	Y	Y	Y	Y	Y	Y	Y	Y	Good (%87.5)
Yilmaz et al. [76]	Y	Y	Y	Y	Y	N	Y	Y	Good (%87.5)
**Madde skoru**	%100	%100	%100	%100	%100	%29.41	%100	%100	

Y, yes; N, no

Questions of JBI Critical Appraisal Checklist for Analytical Cross-sectional Studies

Q1: Were the criteria for inclusion in the sample clearly defined?

Q2: Were the study subjects and the setting described in detail?

Q3: Was the exposure measured in a valid and reliable way?

Q4: Were objective, standard criteria used for measurement of the condition?

Q5: Were confounding factors identified?

Q6: Were strategies to deal with confounding factors stated?

Q7: Were the outcomes measured in a valid and reliable way?

Q8: Was appropriate statistical analysis used?

## Results of the meta-analysis

### Results of the meta-analysis and meta-regression for probably postpartum depression

**Fig. 2:**
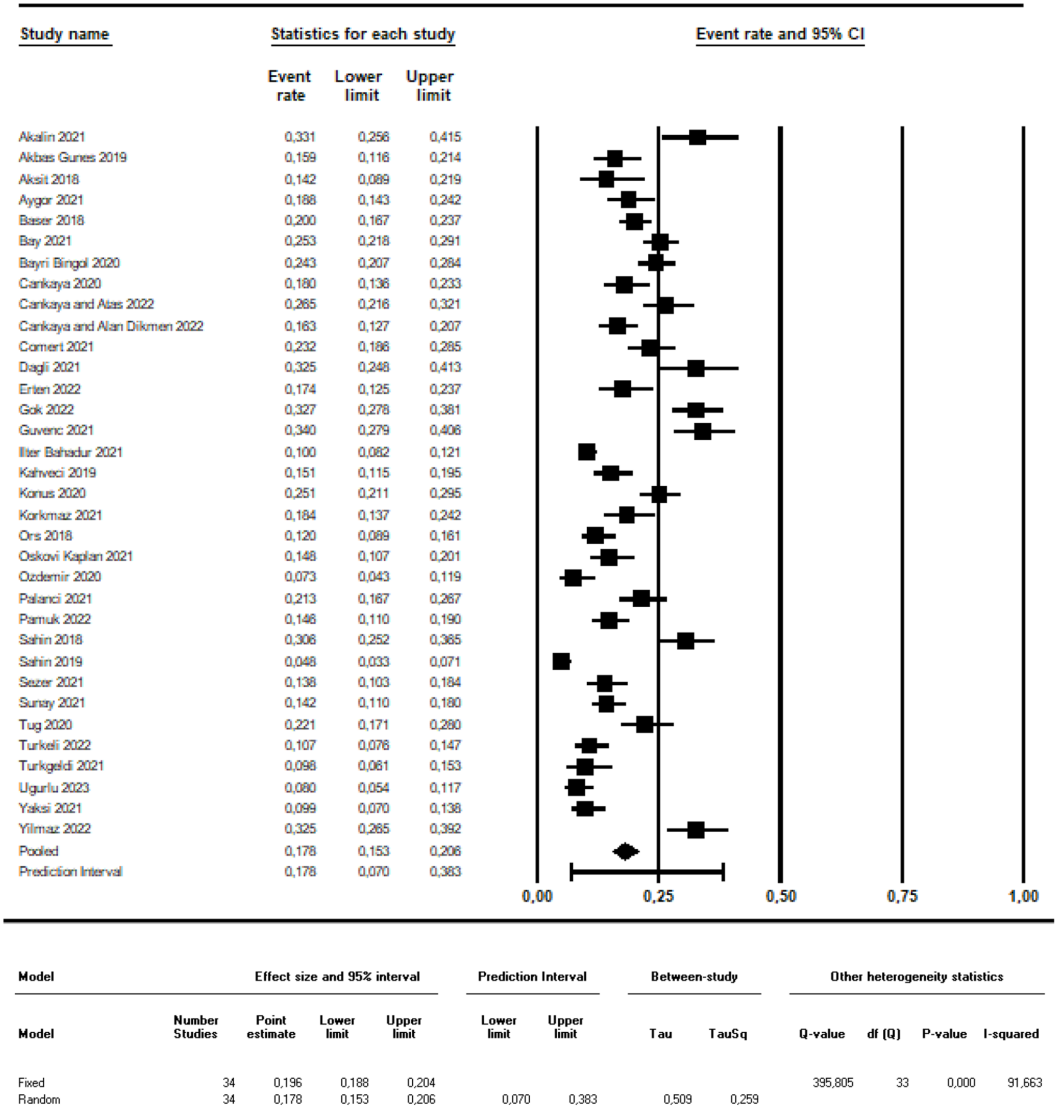
Meta-analysis results on the prevalence of probably postpartum depression

**Fig. 3:**
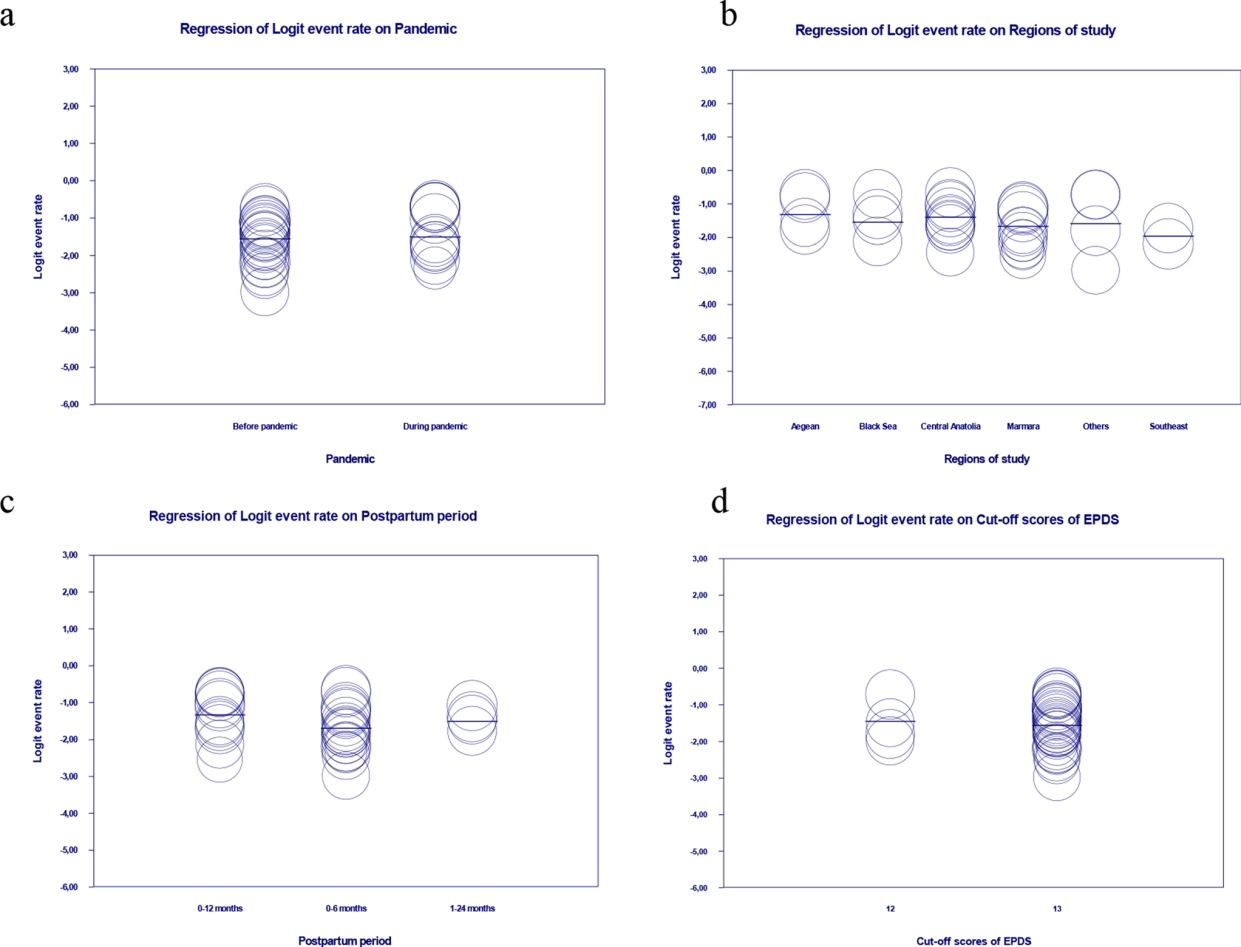
Meta-regressions on the effects of pandemic (**a**), study region (**b**), data collection time (**c**), and data collection tool cut-off value (**d**) on the prevalence of probably postpartum depression

Combined results of 34 studies included in this systematic review and meta-analysis showed that the estimated the prevalence of probably PPD was 17.8% (95% CI: 0.153–0.206; 95% Prediction Interval: 0.070–0.383; I^2^: 91.663; Fig. 2). Besides, meta-regression was made to examine the effects of the region where the studies were conducted, postpartum period when data were collected and the cut-off point for the EPDS on the prevalence of probably PPD. The meta-regression result for each variable (Q = 4.97; df: 5; *p* = 0.420 for the region; Q = 3.74; df: 2; *p* = 0.154 for the postpartum period; Q = 0.04; df: 1; *p* = 0.848 for the cut-off point) and for the model including the variable of the pandemic (Q = 6.89; df: 9; *p* = 0.649; Fig. 3b, c,d) was not statistically significant. Due to the insignificant meta-regression result, subgroup analyses were not made for these variables.

### Subgroup analysis and meta-regression results about the effect of the pandemic on the prevalence of probably postpartum depression

**Fig. 4:**
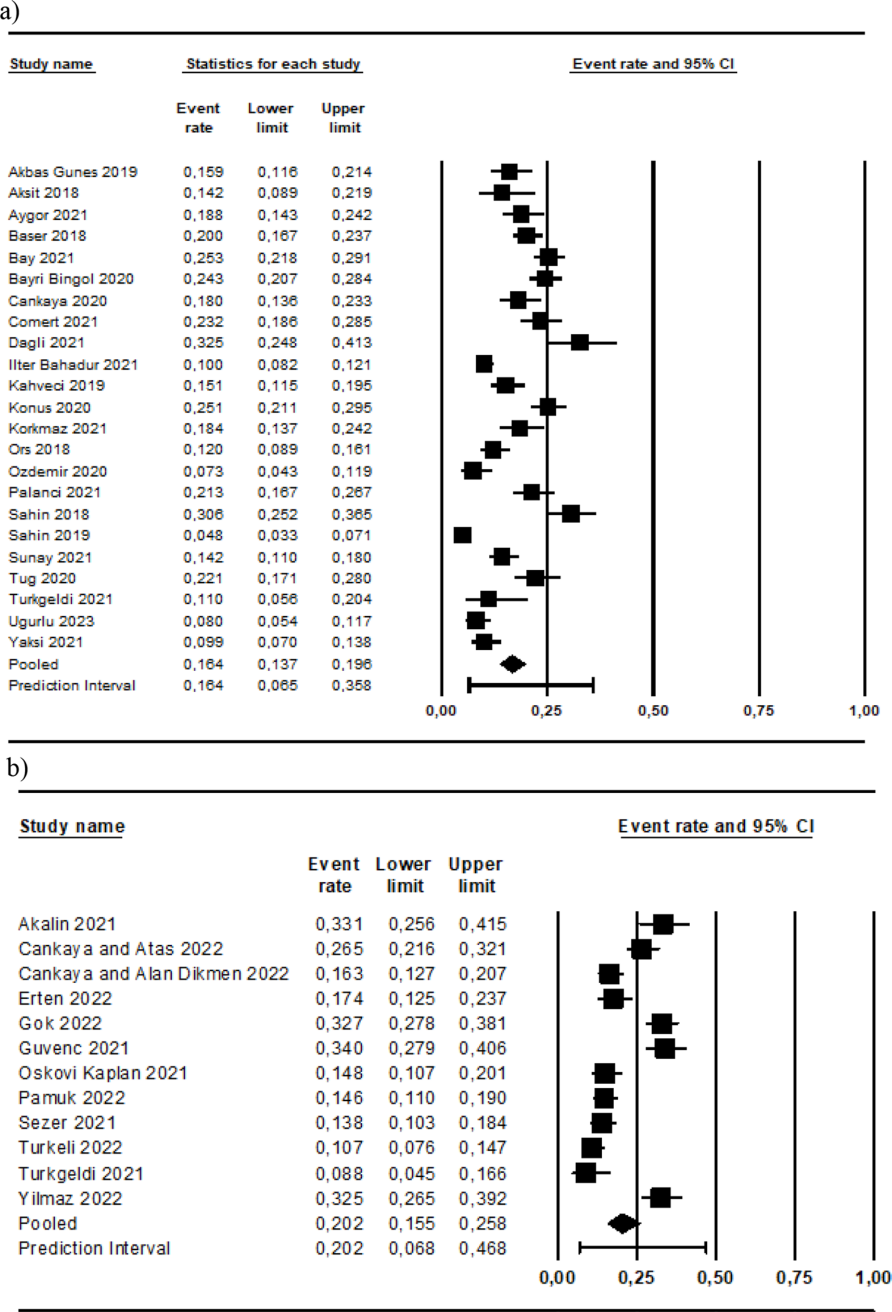
Meta-analysis results on the prevalence of probably postpartum depression before the pandemic (**a**) and during the pandemic (**b**)

According to the subgroup analyses made to determine the effect of the pandemic on the prevalence probably PPD, the estimated prevalence was 16.3% before the pandemic (95% CI: 0.065–0.358; 95% Prediction Interval: 0.065–0.358; I^2^: 91.146; Fig. 4a) and 20.2% during the pandemic (95% CI: 0.068–0.468; 95% Prediction Interval: 0.068–0.468; I^2^: 91.388; Fig. 4b). However, the meta-regression including the moderator variable of the pandemic showed that the difference was insignificant (Q = 1.77; df: 1; *p* = 0.184; Fig. 3a).

### Publication bias of the studies

**Fig. 5:**
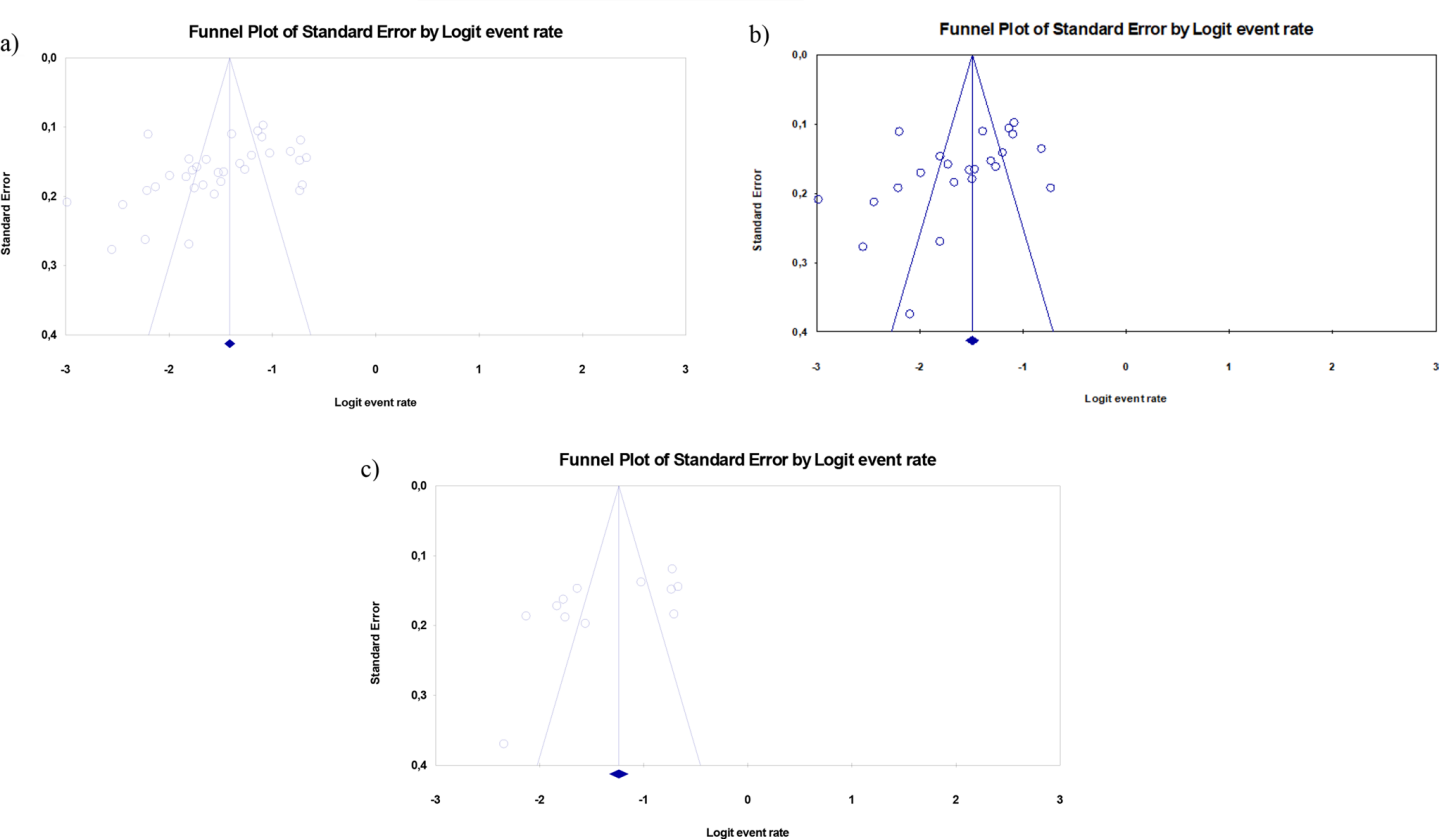
Publication bias about probably postpartum depression. **a**) For total probably postpartum depression. **b**) For before pandemic. **c**) For during pandemic

Funnel plot and Egger’s regression intercept were utilized to examine the publication bias for the total prevalence of probably PPD, the prevalence of probably PPD before the pandemic, and during the pandemic. The publication bias for the prevalence of probably PPD in all the studies (t = 2.954, df: 32, *p* = 0.006), before the pandemic (t = 2.457, df: 21, *p* = 0.023) and during the pandemic (t = 2.350, df: 10, *p* = 0.041) was statistically significant (Fig. 5).

### Factors affecting the development of probably postpartum depression

Out of 34 studies, 32 presented results about the risk factors affecting the development of probably PPD. The present study revealed numerous risk factors related to women and their infants and families (Table 1).

## Discussion

In this systematic review and meta-analysis, the results from 34 cross-sectional studies were combined and presented to determine the prevalence of probably PPD before and during COVID-19 pandemic and the effect of the pandemic on the development of probably PPD. Obtained results are valuable in that they provide comprehensible and updated knowledge about the issue.

In this meta-analysis, based on the results of the cross-sectional studies performed in Turkey before and during the pandemic, the total prevalence of probably PPD (17%) was found to be a lot lower than that reported in prior meta-analyses in Turkey (23.8% and 24%) [18, 19]. However, the total prevalence of probably PPD found in this study is consistent with that in meta-analyses including studies from different countries (11.5- 27% (10, 16, 20–24). These results show that the prevalence of probably PPD is quite common and varies depending on the time and countries in which studies were conducted. In addition, the fact that the prevalence of probably PPD detected in this study is higher than the results of previous studies conducted in Turkey can be explained by the fact that only studies conducted with EPDS were included in this study and the possibility that the prevalence of probably PPD in Turkey has decreased over time.

This study showed that the prevalence of probably PPD (20.2%) during the COVID-19 pandemic was higher than the prevalence of probably PPD (16.3%) in the pre-pandemic period in Turkey, but this difference was not statistically significant. In a meta-analysis including studies performed before the pandemic, Dadi et al. demonstrated that the prevalence of probably PPD was 16.8% [7], which is congruent with the present study. On the other hand, the meta-analyses including studies performed during the pandemic revealed that the prevalence of probably PPD was high compared to that found in the present meta-analysis (26.7-34%). They also showed that the prevalence of probably PPD was comparatively high during the pandemic as compared with the one before the pandemic [2, 4–6], agreeing with the present study. Moreover, in cross-sectional studies in Argentina, China and Saudi Arabia, the prevalence of probably PPD was considerably high during COVID-19 pandemic (37%, 56.9% and 60.7% respectively) as compared with the prevalence of probably PPD at other times [26, 28, 77]. However, Ceulemans et al. [78] and Tsuno et al. [29] reported that the prevalence of probably PPD was lower in cross-sectional studies in Ireland, Norway, Switzerland, the Netherlands, the UK and Japan (13.1% and 13% respectively). Işıkalan et al. [79] also revealed in their study in Turkey that the prevalence of probably PPD was considerably higher in women with COVID-19 than in those without COVID-19 (53.3% versus 13.3%). These findings are important in that they show COVID-19 pandemic for probably PPD.

In the present study, the prevalence of probably PPD did not differ in terms of the regions where the studies were conducted, which is conflicting with evidence from a prior meta-analysis made in Turkey [18] and a study performed by Tolossa et al. [23] in Etiopia. Indeed, Tolossa et al. [23] reported regional differences in the prevalence of probably PPD (12.20% versus 33.82%). Liu et al. [20] showed in their meta-analysis that the prevalence of probably PPD changed from country to country and was high in developing countries, especially China.

The results of the current meta-regression showed that the postpartum period when the data were gathered (0–6 months, 0–12 months and 1–24 months) and the cut-off point for the EPDS (≥ 12 and ≥ 13) were not effective moderator variables in the prevalence of probably PPD. However, Gao et al. [5], found in their meta-analysis that the prevalence of probably PPD varied with different cut-off points for the EPDS and the postpartum period when data were obtained. Besides, a cross-sectional study using cut-off values of the EPDS at large intervals demonstrated that the prevalence of probably PPD varied with the cut-off values in Japan (28.7% for the cut-off point of ≥ 9, which is frequently used in Japan; 18.6% for ≥ 11 and 13.1% for ≥ 13) [29]. It can be suggested that using cut-off values of the scales whose validity and reliability have been achieved in a given country and the postpartum period when data are gathered are of great importance.

The present study showed that there were many maternal, infant and familial risk factors that increased the prevalence of probably PPD, comparable with the findings from other meta-analyses in Turkey and many other countries [4, 7, 16, 18, 20, 22, 23, 25–27, 29]. The evidence mentioned above is important since it suggests that probably PPD is a multifaceted mental problem.

### Strengths and limitations of the study

The strengths of this meta-analysis are that the studies included were retrieved from a wide variety of databases, were conducted in different regions of Turkey and had the latest and high evaluation scores of their quality. Besides, data were collected with the same tool, i.e. EPDS, in all the studies, the total sample size was large (*n* = 10 236) and multiple analyses including subgroup analyses and meta-regression were used, all of which helped to obtain high-quality results. However, high heterogeneity among the studies detected in all the meta-analyses and significant publication bias of the studies can be considered as limitations likely to decrease the strength of the present study. To keep high heterogeneity under control, the random effect model was adopted in all the meta-analyses; the calculated point estimates were reported with 95% CI and prediction interval values. This study was planned to include studies in which PPD screening was performed between 1 and 12 months postpartum. However, during the selection process of the studies, it was seen that some studies collected data from women between 1 and 24 months postpartum and some from women within the first month, and the sample group of this study was updated to women between 0 and 24 months postpartum. Therefore, the effect of the time when screening was performed on the effect size was evaluated by meta-regression.

## Conclusion and recommendations

This meta-analysis revealed that the total prevalence of probably PPD in Turkey was 17.8 and that the prevalence of probably PPD (20.2%) during COVID-19 pandemic was higher compared to that before the pandemic (16.3%). Also, many maternal-infant and familial risk factors were shown to be effective in probably PPD, consistent with the evidence in the literature. Besides, this meta-analysis demonstrated that the prevalence of probably PPD in Turkey did not vary with geographical regions, the postpartum periods when data were collected and the cut-off points of the EPDS.

In light of the results of this meta-analysis, it can be recommended that health professionals should pay attention to risk factors of probably PPD during perinatal follow-up and care, take preventive measures, perform early screenings and refer women at high risk to specialized health centers for further diagnosis and treatment. In addition, administrators and educators working in the healthcare sector should integrate the results of current studies into formulation and implementation of policies and provision of healthcare regarding probably PPD. Moreover, similar meta-analyses could be carried to confirm the results of this meta-analysis. Qualitative studies could be performed to examine experiences of the women with PPD and experimental studies could conducted to reduce the risk of PPD and improve the management of PPD.

## Electronic supplementary material

Below is the link to the electronic supplementary material.

Supplementary Material 1

## Data Availability

No datasets were generated or analysed during the current study.
