# Supplementary material for: Comparison of the prevalence of probably postpartum depression before and during the covid-19 pandemic in Turkey: a systematic review and meta-analysis
Source: Soc Psychiatry Psychiatr Epidemiol. 2025 Apr 23;60(10):2469–85. doi: 10.1007/s00127-025-02905-4 (PMC12449347; doi:10.1007/s00127-025-02905-4)
Supplement: Supplementary file 1 — Supplementary Material 1 [file 127_2025_2905_MOESM1_ESM.docx]

**TABLE 1:** Search strategy.

| **Database** | **Search terms** | **Articles** |
| --- | --- | --- |
| PubMed | (((postpartum depression) ) OR (postnatal depression)) AND (Turkey) | 290 |
| EbscoHost Databases (Emerald Premier eJournal,  ScienceDirect Freedom Collection, Scopus  SpringerLink \| Palgrave \| Adis Journals, Nature \| Academic Journal, Taylor & Francis  MEDLINE: PubMed,  Web of Science  Wiley Online Library,  TR Dizin, DynaMed) | postpartum depression or postnatal depression and Turkey | 5.381 |
| OVID Journals (ULAKBIM-Ekual) | ((postpartum depression or postnatal depression) and (Turkey).mp. [mp=title, abstract, full text, caption text] | 88 |
| Science Direct | (postpartum depression or postnatal depression) and (Turkey) | 308 |
| Web of Science | (postpartum depression or postnatal depression) and (Turkey) | 305 |
| [ULAKBIM Databases](https://kutuphane.adu.edu.tr/vt.asp#collapse1454) | (postpartum depression or postnatal depression) and (Turkey) | 1967 |
| DergiPARK | (title: "postpartum depresyon" OR title: "postnatal depresyon" OR title: "doğum sonrası depresyon" ) AND (pubyear: (>=2017 AND <=2023)) | 27 |
| TR Dizin | (postpartum depression or postnatal depression) and (Turkey) | 0 |
| YÖK-Natural Thesis Centre | (postpartum depression or postnatal depression) | 26 |

**TABLE 1:** Some descriptive characteristics of women participating in the studies.

| **Authors (year)** | **Education level,**  **n (%)** | **Employment status, n (%)** | **Perceived income level, n (%)** | **Number of pregnancies,**  **n (%)** | **Mode of birth,**  **n (%)** | **Rural or urban area, n (%)** | **Planning status of pregnancy,**  **n (%)** |
| --- | --- | --- | --- | --- | --- | --- | --- |
| Akalin et al. [61] | High school and below: 47 (35.3%)  University and above: 86 (64.7%) | Employed: 76 (57.1%)  Housewife: 57 (42.9%) | Poor: 50 (37.6%)  Moderate: 62 (46.6%)  Good: 21 (15.8%) | No information. | Vaginal: 45 (33.8%)  Caesarean: 88 (66.2%) | Rural: 0  Urban: 133 (100%) | No information. |
| Akbas Gunes, [51] | Primary and secondary school: 60 (27.3%)  High school: 80 (36.4%)  University: 80 (36.4%) | Employed: 80 (36.4%)  Housewife: 140 (%63.6%) | Poor: 15 (6.8%)  Moderate: 200 (90.9%)  Good: 5 (2.3%) | 1: 90 (40.9%)  2-3: 95 (43.1%)  4 and above: 41 (16%) | No information. | Rural: 0  Urban: 220 (100%) | No information. |
| Aksit, [42] | Primary and secondary school: 5 (4.4%)  High school: 12 (10.6%)  University: 96 (85.0%) | Employed: 81 (71.7%)  Housewife: 32 (28.3%) | Poor: 14 (12.4%)  Moderate: 29 (25.7%)  Good: 70 (61.9%) | No information. | Vaginal: 31 (27.4%)  Caesarean: 82 (72.6%) | Rural: 0  Urban: 113 (100%) | Planned: 92 (81.4%)  Unplanned: 21 (18.6%) |
| Aygor and Metin, [52] | Literate-Primary and secondary school: 24 (10.0%)  High school: 119 (49.6%)  University: 97 (40.4%) | Employed: 75 (31.3%)  Housewife: 165  (68.7%) | Poor: 129 (53.8%)  Moderate: 37 (15.4%)  Good: 74 (30.8%) | 1: 97 (40.4%)  2 and above: 143 (59.6%) | Vaginal: 105 (43.8%)  Caesarean: 135 (56.2%) | Rural: 0  Urban: 240 (100%) | Planned: 204 (85.0%)  Unplanned: 36 (15.0%) |
| Baser, [43] | Primary and secondary school: 78 (15.3%)  High school: 166 (32.5%)  University: 267 (52.2%) | Employed: 165 (32.3%)  Housewife: 346 (67.7%) | No information. | No information. | Vaginal: 169 (33.1%)  Caesarean: 342 (66.9%) | Rural: 0  Urban: 511 (100%) | Planned: 403 (78.8%)  Unplanned: 108 (21.2%) |
| Bay and Sayiner, [53] | No information. | No information. | Poor: 101 (18.40%)  Moderate: 362 (65.80%)  Good: 87 (15.80%) | No information. | Vaginal: 321 (58.40%)  Caesarean: 229 (41.60%) | Rural: 0  Urban: 550 (100%) | No information. |
| Bayri Bingol and Demiroz Bal, [44] | Secondary school and below: 177 (36.8%)  High school and above: 304 (63.2%) | Employed: 124 (6.4%)  Housewife: 346 (3.6%) | Poor: 79 (16.5%)  Moderate: 338 (70.4%)  Good: 63 (13.1%) | No information. | Vaginal: 295 (62.1%)  Caesarean: 180 (37.8%) | Rural: 0  Urban: 475 (100%) | Planned: 329 (68.5%)  Unplanned: 151 (31.5%) |
| Cankaya, [54] | Primary and secondary school-High school: 95 (38.8%)  University: 150 (61.2%) | Employed: 42 (17.1%)  Housewife: 203 (82.9%) | Poor: 27 (11.1%)  Moderate: 152 (62.0%)  Good: 66 (26.9%) | 1: 184 (75.1%)  2: 33 (13.5%)  3 and above: 18 (7.4%) | Vaginal: 213 (86.9%)  Caesarean: 32 (13.1%) | Rural: 64 (26.1%)  Urban: 181 (73.9%) | Planned: 199 (81.2%)  Unplanned: 46 (18.8%) |
| Cankaya and Alan Dikmen, [55] | Primary and secondary school: 140 (41.5%)  High school: 139 (41.3%)  University: 58 (17.2%) | Employed: 82 (24.3%)  Housewife: 255 (75.7%) | Poor: 9 (2.7%)  Moderate: 190 (56.4%)  Good: 138 (41.9%) | 1: 151 (44.8%)  2: 96 (28.5%)  3 and above: 90 (26.7%) | No information. | Rural: 0  Urban: 337 (100%) | No information. |
| Çankaya and Ataş, [56] | Primary and secondary school-High school: 190 (70.9%)  University: 78 (29.1%) | Employed: 86 (32.1%)  Housewife: 182 (67.9%) | Poor: 33 (12.3%)  Moderate: 182 (67.9%)  Good: 53 (19.8%) | 1: 105 (39.2%)  2: 87 (32.5%)  3 and above: 76 (28.3%) | Vaginal: 166 (61.9%)  Caesarean: 102 (38.1%) | Rural: 0  Urban: 268 (100%) | Planned: 214 (79.9%)  Unplanned: 54 (20.1%) |
| Comert and Bayri Bingol, [45] | No information. | Employed: 38 (13.6%)  Full‐time Housewife: 242 (86.4%) | Poor: 77 (27.5%)  Moderate: 199 (71.4%)  Good: 4 (1.1%) | No information. | Vaginal: 169 (60.4%)  Caesarean: 111 (39.6%) | Rural: 0  Urban: 280 (100%) | No information. |
| Dagli et al. [71] | Primary school: 26 (21.1%)  Secondary school and above: 97 (78.9%) | Employed: 63 (51.2%)  Housewife: 60 (48.8%) | Poor: 32 (26.0%)  Moderate-Good: 91 (74.0%) | No information. | Vaginal: 57 (46.3%)  Caesarean: 66 (53.7%) | Rural: 0  Urban: 123 (100%) | No information. |
| Erten et al. [65] | Primary and secondary school: 76 (51.7%)  High school: 41 (27.9%)  University: 30 (20.4%) | Employed: 16 (10.9%)  Housewife: 131 (89.1%) | Poor: 13 (8.8%)  Moderate: 95 (64.6%)  Good: 39 (26.5%) | No information. | Vaginal: 64 (43.5%)  Caesarean: 83 (56.5%) | Rural: 0  Urban: 147 (100%) | No information. |
| Gok et al. [66] | Primary and secondary school: 38 (11.9%)  High school: 202 (63.5%)  University: 78 (24.5%) | Employed: 196 (621.6%)  Housewife: 122 (38.4%) | Poor: 168 (52.8%)  Moderate: 125 (39.3%)  Good: 25 (7.9%) | 1: 95 (29.9%)  2-3: 203 (63.8%)  4 and above: 20 (6.3%) | Vaginal: 158 (55.7%)  Caesarean: 160 (44.3%) | Rural: 78 (24.5)  Urban: 240 (75.5%) | Planned: 287 (90.3%)  Unplanned: 31 (9.7%) |
| Guvenc et al. [57] | Primary and secondary school: 37 (17.5%)  High school: 61 (28.8%)  University: 114 (53.8%) | Employed: 79 (37.3%)  Housewife: 133 (62.7%) | No information. | No information. | Vaginal: 102 (48.1%)  Caesarean: 110 (51.9%) | Rural: 0  Urban: 212 (100%) | No information. |
| Ilter Bahadur et al. [69] | Literate and illiterate: 334 (37.1%)  Primary and secondary school: 428 (47.6%)  High school: 104 (11.6%)  University: 34(3.8%) | Employed: 23(2.6%)  Housewife: 877 (97.4%) | Poor: 854 (94.9%)  Moderate-Good: 46 (5.1%) | No information. | No information. | Rural: 0  Urban: 900 (100%) | Planned: 832 (92.4%)  Unplanned: 68 (7.6%) |
| Kahveci, [70] | No information. | Employed: 10 (3.2%)  Housewife: 301 (96.8%) | Poor: 83 (26.7%)  Moderate-Good: 228 (73.3%) | No information. | Vaginal: 174 (55.9%)  Caesarean: 137 (44.1%) | Rural: 92 (29.5%)  Urban: 219 (70.5%) | Planned: 213 (68.5%)  Unplanned: 98 (31.5%) |
| Konus, [46] | Primary and secondary school: 58 (14.1%)  High school: 208 (50.5%)  University: 146 (35.4%) | Employed: 206 (50.0%)  Housewife: 206 (50.0%) | Poor: 17  (4.1%)  Moderate: 255 (61.9%)  Good: 120 (29.1%) | 1: 243 (60.0%)  2-3: 159 (38.6%)  4 and above: 10 (2.4%) | Vaginal: 290 (70.4%)  Caesarean: 122 (29.6%) | Rural:0  Urban: 412 (100.0%) | Planned: 301 (73.1%)  Unplanned: 111 (26.9%) |
| Korkmaz and Yilar Erkek, [62] | Primary and secondary school: 87 (42%)  High school: 62 (30.0%)  University: 58 (28.0%) | Employed: 44 (21.3%)  Housewife: 163 (78.7%) | Poor: 58 (28.0%)  Moderate: 119 (57.5%)  Good: 30 (14.5%) | No information. | No information. | Rural: 0  Urban: 207 (100.0%) | No information. |
| Ors, [47] | Literate and illiterate: 10 (3.1%)  Primary and secondary school: 140 (43.2%)  High school: 91 (28.1%)  University: 83 (25.6%) | Employed: 80 (24.7%)  Housewife: 244 (75.3%) | Poor: 121 (37.3%)  Moderate: 171 (52.8%)  Good: 32 (9.9%) | No information. | Vaginal: 86 (26.5%)  Caesarean: 238 (73.5%) | Rural: 0  Urban: 324 (100.0%) | Planned: 272 (84.0%)  Unplanned: 52 (16.0%) |
| Oskovi Kaplan et al. [58] | Literate-Primary and secondary school: 81 (36.3%)  High school: 119 (53.4%)  University: 23 (10.3%) | No information. | No information. | No information. | Vaginal: 123 (51.1%)  Caesarean: 100 (48.9%) | Rural: 0  Urban: 223 (100.0%) | No information. |
| Ozdemir, [48] | Literate and illiterate: 17 (8.8%)  Primary and secondary school: 100 (51.8%)  High school: 33 (17.1%)  University: 43 (22.3%) | Employed: 36 (18.7%)  Housewife: 157 (81.3%) | Poor: 47 (24.6%)  Moderate: 130 (68.1%)  Good: 14 (7.3%) | 1: 63 (32.6%)  2: 54 (28.0%)  3 and above: 76 (39.4%) | Vaginal: 87 (45.1%)  Caesarean: 106 (54.9%) | Rural: 0  Urban: 193 (100.0%) | Planned: 129 (66.8%)  Unplanned: 64 (33.2%) |
| Palancı and Aktaş, [63] | Primary and secondary school: 84 (31.3%)  High school: 77 (30.3%)  University: 93 (36.6%) | Employed: 77 (30.3%)  Housewife: 177 (69.7%) | No information. | No information. | Vaginal: 142 (55.9%)  Caesarean: 112 (44.1%) | Rural: 0  Urban: 254 (100.0%) | Planned: 201 (79.1%)  Unplanned: 53 (20.9%) |
| Pamuk and Guclu, [67] | Literate and illiterate-Primary and secondary school: 148 (49.0%)  High school-University: 154 (51.0%) | Employed: 81 (26.5%)  Housewife: 221 (73.5%) | No information. | No information. | Vaginal: 117 (38.7%)  Caesarean: 185 (61.3%) | Rural: 0  Urban: 302 (100.0%) | Planned: 229 (75.8%)  Unplanned: 73 (24.2%) |
| Sahin, [68] | Literate and illiterate: 18 (7.0%)  Primary and secondary school: 121 (57.5%)  High school: 60 (23.5%)  University: 56 (22.0%) | Employed: 59 (23.2%)  Housewife: 196 (76.9%) | Poor: 119 (46.6%)  Moderate: 127 (49.8%)  Good: 9 (3.6%) | No information. | Vaginal: 96 (37.6%)  Caesarean: 159 (62.3%) | Rural: 0  Urban: 255 (100.0%) | Planned: 215 (84.3%)  Unplanned: 40 (15.7%) |
| Sahin and Seven, [72] | Literate and illiterate: 48 (9.7%)  Primary and secondary school: 252 (50.7%)  High school: 118 (23.7%)  University: 79 (15.9%) | Employed: 74 (14.9%)  Housewife: 423 (85.1%) | No information. | No information. | Vaginal: 311 (62.6%)  Caesarean: 186 (37.4%) | Rural: 0  Urban: 497 (100.0%) | No information. |
| Sezer, [49] | Literate and illiterate: 10 (3.6%)  Primary and secondary school: 76 (27.0%)  High school: 89 (31.6%)  University: 107 (37.6%) | Employed: 99 (35.1%)  Housewife: 183 (64.9%) | Poor: 133 (47.2%)  Moderate: 100 (35.4%)  Good: 49 (17.4%) | 1: 98 (34.8%)  2: 110 (39.0%)  3 and above: 74 (26.2%) | Vaginal: 94 (33.3%)  Caesarean: 188 (66.7%) | Rural: 0  Urban: 282 (100.0%) | Planned: 184 (65.2%)  Unplanned: 98 (34.8%) |
| Sunay et al. [73] | Literate and illiterate: 30 (7.9%)  Primary and secondary school: 107 (28.1%)  High school: 191 (50.1%)  University: 53 (13.9%) | Employed: 31 (8.1%)  Housewife: 350 (91.9) | Poor: 78 (20.5%)  Moderate: 278 (73.0%)  Good: 25 (6.5%) | 1: 106 (27.8%)  2 and above: 275 (72.2%) | No information. | Rural: 0  Urban: 381 (100%) | No information. |
| Tug, [59] | Literate and illiterate: 7 (3.2%)  Primary and secondary school: 78 (35.2%)  High school: 66 (29.7)  University: 71 (32.0%) | Employed: 64 (28.8%)  Housewife: 158 (71.2%) | Poor:  Moderate:  Good: | 1: 72 (32.4%)  2-3: 110 (49.5%)  4 and above: 40 (18.2%) | Vaginal: 109 (49.1%)  Caesarean: 113 (50.9%) | Rural: 0  Urban: 222 (100%) | Planned: 173 (77.9%)  Unplanned: 49 (22.1%) |
| Turkeli, [64] | Primary and secondary school: 57 (19.0%)  High school: 166 (55.3%)  University: 77 (25.7%) | Employed: 42 (14.0%)  Housewife: 258 (86.0%) | Poor: 22 (7.4%)  Moderate: 214 (71.3%)  Good: 64 (21.3%) | No information. | No information. | Rural:  Urban: 300 (100%) | Planned: 228 (76.0%)  Unplanned: 72 (24.0%) |
| Turkgeldi and Yildiz, [41] | No information. | No information. | No information. | No information. | Vaginal: 47 (28.7%)  Caesarean: 117 (71.3%) | Rural: 0  Urban: 164 (100%) | No information. |
| Ugurlu et al. [60] | Primary and secondary school: 91 (30.3%)  High school: 120 (40.0%)  University: 89 (29.7%) | Employed: 77 (25.7%)  Housewife: 223 (74.3%) | Poor: 57 (19.0%)  Moderate: 209 (69.7%)  Good: 34 (11.3%) | 1: 125 (41.7%)  2: 105 (35.0%)  3 and above: 70 (23.3%) | Vaginal: 140 (46.7%)  Caesarean: 160 (53.3%) | Rural: 0  Urban: 300 (100%) | No information. |
| Yaksi and save, [50] | High school and below: 160 (52.8%)  University and above: 143 (47.2%) | Employed: 146  Housewife: 157 | No information. | No information. | Vaginal: 91 (30.0%)  Caesarean: 212 (70.0%) | Rural: 0  Urban: 303 (100%) | Planned: 241 (79.5%)  Unplanned: 62 (20.5%) |
| Yilmaz et al. [74] | Primary and secondary school: 33 (16%)  High school: 40 (19.4%)  University and above: 133 (64.6%) | Employed: 61 (29.6)  Housewife: 145 (70.4) | No information. | No information. | Vaginal: 74 (35.9)  Caesarean: 132 (64.1) | Rural: 0  Urban: 206 (100%) | No information. |
